# Comparison of visual outcomes and optical aberrations after SMILE with intraoperative Kappa angle adjustments between small and large Kappa angles

**DOI:** 10.1038/s41598-024-65366-w

**Published:** 2024-06-24

**Authors:** Xiaojuan Lai, Xi Liu, Tao Zeng, Yi Huang, Xin Yang

**Affiliations:** 1 Department of Ophthalmology, The Second People’s Hospital of Yibin, Yi’bin, 644000 China; 2 The University of Alabama, Tuscaloosa, AL 35487 USA

**Keywords:** Eye diseases, Medical research

## Abstract

This study compares postoperative visual outcomes and optical aberrations after Small Incision Lenticule Extraction (SMILE) in patients with both small (S-Kappa: Kappa angle < 0.2 mm) and large Kappa (L-Kappa: Kappa angle ≥ 0.2 mm) angles. The evaluated aberrations include total higher-order aberrations (HOAs), horizontal coma (HC), vertical coma (VC), and spherical aberrations (SA), with procedures incorporating intraoperative Kappa angle adjustments. We retrospectively analyzed patient records undergoing SMILE utilizing linear mixed models (LMM). We assessed adjusted mean uncorrected distance visual acuity (UDVA), Strehl ratio (SR), total HOAs, VC, and SA at pupils of 3 mm and 6 mm for both S-Kappa and L-Kappa. The disparities between S-Kappa and L-Kappa were evaluated by LMM's adjusted mean differences. The differences in optical metrics were also assessed in eyes grouped by myopia levels: low, moderate, and high. A sensitivity analysis was conducted on a threshold of Kappa angle at 0.3 mm. Eight-five patients (169 eyes) were analyzed, and no significant pre-operative difference was found in UDVA (*p* = .222) or spherical equivalent (*p* = .433). Post-operative differences were found in SR at 3 mm pupil size (−0.06, *p* = .022), total HOA 3 mm (0.15, *p* = .022), HC 3 mm (0.04, *p* = .042), VC 3 mm and 6 mm (−0.08, *p* = .041; 0.04, *p* = .041). The stratified analysis for high myopia revealed significant differences in UDVA (−0.04, *p* = .037), HC 3 mm (0.07, *p* = .03), VC 6 mm (−0.21, *p* = .001), and SA 3 mm and 6 mm (0.07, *p* = .037; −0.09, *p* = .037). Sensitivity analysis showed no significant difference using a 0.3 mm Kappa threshold. While some optical aberrations exhibited statistical differences between S-Kappa and L-Kappa, their clinical significance is limited. Thus, a large Kappa angle might not substantially influence post-operative optical aberrations when intraoperative Kappa angle adjustments are implemented.

## Introduction

Myopia, also known as nearsightedness, is a refractive error that leads to blurred vision when looking at distant objects^1^. Myopic astigmatism, on the other hand, is a combined refractive error where individuals experience blurred vision at all distances due to irregularities in the corneal shape^2^. These refractive errors have a significant impact on an individual's quality of life, emphasizing the importance of effective treatment options^3^. Myopia is a global health concern, with estimates suggesting that nearly half of the world's population will be affected by 2050^1^. Concurrently, the prevalence of myopic astigmatism is also on the rise, adding to the burden of vision impairment globally^4^.

Given the challenges posed by these refractive errors, several treatment options have emerged over the years. Among them, the femtosecond laser-assisted Small Incision Lenticule Extraction (SMILE) stands out as a promising solution. SMILE is a novel refractive surgical procedure that has gained popularity due to its minimally invasive approach, high efficacy, and fewer complications compared to other laser refractive surgeries^5,6^. The procedure works by using a femtosecond laser to create a lenticule within the cornea, which is then extracted through a small incision. This approach changes the corneal shape, thereby correcting refractive errors^7^. Research confirms that SMILE is an effective treatment for myopia and myopic astigmatism, boasting reliable predictability, efficacy, stability, and safety in its corrective capabilities. Furthermore, this approach consistently results in high levels of patient satisfaction^6,8,9^. However, despite its many benefits, SMILE outcomes can be influenced by several factors, including the Kappa angle.

While SMILE offers numerous advantages, it's important to note that its outcomes aren't uniform for all patients. Factors such as the Kappa angle play a pivotal role in the post-operative results. In refractive surgery, the Kappa angle, the deviation between the visual and pupillary axes, is pivotal for aligning the visual axis. If misaligned, it can influence postoperative Higher-Order Aberrations (HOAs)^10,11^. HOAs are complex optical flaws affecting visual clarity, with types like coma aberrations, which can smear point-source light images, and spherical aberration (SA), causing peripheral light rays to refract differently than central ones^12^. The Strehl ratio (SR), gauging optical image quality, shows a perfect system at a value of 1, with anything lower indicating aberrations^13^. Monitoring these metrics before and after refractive surgeries is essential for predicting outcomes and ensuring visual quality. Prior research indicates that patients with larger kappa angles may be predisposed to eccentric ablation. This phenomenon could lead to an increase in postoperative HOAs, consequently reducing the overall quality of vision^14–17^.

While a significant body of literature has explored the disparities between SMILE and established refractive surgical techniques like Laser-Assisted In Situ Keratomileusis (LASIK) and Laser-Assisted Sub-Epithelial Keratomileusis (LASEK)^18–21^, limited research has probed the differential outcomes of SMILE surgery based on Kappa angles' size. This is particularly noteworthy given the potential nuances introduced by varying degrees of myopia. Centering deviation in myopic surgery is a common concern, with previous studies indicating that deviations exceeding 0.2 mm can affect postoperative outcomes^14,22,23^. Historically, excimer laser surgery utilized corneal topography-guided centering throughout the procedure, ensuring continuous tracking. However, femtosecond laser surgery lacks this guidance mode. Some scholars argue that patients with larger Kappa angles are unsuitable for SMILE surgery due to this limitation^22^. Nonetheless, we contend that current femtosecond laser centering techniques, including watermark-centered alignment, corneal light reflection method, and manual Kappa adjustments, allow for precise centering without compromising surgical efficacy. Recognizing this knowledge gap, our study aims to compare outcomes between small and large Kappa angle groups following SMILE surgery with Kappa adjustment in a cohort of patients diagnosed with myopia and myopic astigmatism. Our retrospective study aimed to analyze whether there is a significant difference in the optical metrics between S-Kappa and L-Kappa groups while investigating the effectiveness of these centering methods.

## Materials and methods

### Study subjects

The study was conducted with the tenets of the Declaration of Helsinki and was approved by the Institutional Review Board of The Second People’s Hospital of Yibin (approval number: 2020-152-01). Before the surgery, all participants were given and signed informed consent. This retrospective comparative study examined 85 patients (169 eyes) diagnosed with myopia and myopic astigmatism. These patients underwent SMILE procedures between October 10^th^, 2020, and September 1^st^ 2022 at the Department of Ophthalmology Center The Second People’s Hospital of Yibin, Yibin City, China. Inclusion criteria are 18–40 years old, pre-operative Mean Spherical Equivalent (MSE) is between -8.75 Diopter (D) and -0.75D, and the corneal morphology is normal, clear, and without opacity or specks. Soft contact lenses were discontinued for more than one week, rigid contact lenses were discontinued for more than one month, and orthokeratology lenses were discontinued for more than three months. Patients with intraocular diseases, history of eye trauma or surgery, pregnancy, and connective tissue or autoimmune diseases were excluded. Before conducting this study, routine postoperative examinations were performed 1 day, 1 week, 1 month, and 3 months after SMILE surgery. Visual metrics showed stability primarily at one month. Therefore, we limited data collection to the one-month follow-up visit. These examinations included visual acuity, intraocular pressure, refraction, and slit lamp evaluation. However, corneal topography was not routinely checked during these follow-up visits. The corneal topography was specifically examined for this study. In the study, SR, HOA, visual clarity (VC), and SA required corneal topography assessment to obtain results. All surgeries were performed by the same surgeon (Dr. Xi Liu), and all collected data were from more than one month postoperatively.

### Examination methods

All cases underwent routine preoperative examinations for refractive surgery: visual acuity, non-contact tonometry, slit lamp microscopy, computerized refraction, comprehensive refraction, dilated refraction, dominant eye judgment, corneal ultrasonography, Pentacam anterior segment analyzer (Model HR, Oculus, Germany), and Topolyzer examination, etc. Among these, the results from the Pentacam anterior segment analyzer, with good repeatability and high image quality, were selected for analysis: the preoperative pupillary center coordinates relative to the corneal vertex were recorded as the Kappa angle coordinates, and the size of the Kappa angle = $\sqrt{{x}^{2}+{y}^{2}}$.

### Surgical technique

The VisuMax femtosecond laser system (Carl Zeiss, Germany) was used for the SMILE procedure. During the surgery, patients were instructed to fix their gaze on the target green light, adjust the watermark to the center, and manually adjust and confirm based on the preoperative Pentacam anterior segment analyzer's Kappa angle data. Vacuum suction was initiated and maintained until the scanning was completed. The laser pulse frequency was 500 kHz, with a spot distance of 4.5 μm, a spot diameter of 4.5 μm, a corneal cap thickness of 120 μm, a corneal cap diameter of 7.5 mm, a lenticule diameter of 6.5 mm, a base thickening of 10 μm, a 2 mm wide incision was made on the top of the cornea, at an angle of 135°, and both the lenticule and the small incision side-cut angles were 90°. After scanning was completed, a microseparator was used to lift the edge of the separated corneal cap, further separating the upper and lower surfaces of the internal lenticule, and then the lenticule was removed with microforceps. The intraoperative Kappa angle adjustment was implemented as described by Shao et al.^14^.

### Data collection and variables

The preoperative assessment comprised a slit-lamp examination, dilated fundus evaluation, uncorrected distance visual acuity (UDVA) determination, refraction measurement, and Pentacam examinations. The same parameters, including higher-order aberrations, refraction, and UDVA, were also collected one month after surgery. WaveScan measurements were performed before surgery and at the one-month postoperative follow-up. These assessments were conducted in a darkened room. Before the aberrometry scan, patients were advised to blink several times to reduce the tear film's impact on the results. The image capture was completed within three seconds. All images were captured by a single skilled technician, and the average value derived from three high-quality images was used for the analysis. To focus on the period of greatest measurement stability, data were collected from patients at a one-month follow-up appointment. Due to low patient compliance at the three-month follow-up, data from that time point were excluded from this study.

Given the variations in pupil sizes during each measurement, both within the same patient and between different patients, as well as the significant correlation between pupil sizes and aberrations, wavefront aberrations were computed using the Optical Society of America's notation. Root mean square of total HOA, horizontal coma (HC), VC, SA, and SR were documented at pupil sizes of 3 mm and 6 mm. UDVA is converted to a logarithm of the minimum angle of resolution (LogMAR) units. Based on the Kappa angles, eyes were grouped as small Kappa angle (< 0.2 mm) and large Kappa angle group ($\ge$ 0.2 mm). Based on the pre-operative MSE, all patients were categorized into three groups using standard diagnostic classification: low myopia with MSE > -3.0 diopters (D), moderate myopia (-6.0 D < MSE ≤ -3.0 D), and high myopia (MSE ≤ -6.0 D)^4,24^. This classification and diagnosis of myopia is commonly used and well documented^25–28^. The MSE is calculated by spherical component (S) plus half of the cylindrical component (C), which were determined by phoropters.

### Statistical analysis

Before enrolling patients and collecting data, we conducted a sample size calculation and power analysis using the R package “pwr.” Specifically, to achieve 80% statistical power, approximately 100 eyes were required to detect a minimum Cohen’s D effect size of 0.2 (considered small). As a result, our study achieved over 95% power to detect a minimum effect size of 0.2 with a total of 169 eyes. Due to the multilevel nature of the data, linear mixed models were constructed to assess the adjusted means, or “Least square means”, and their difference between small and large Kappa angle groups, accounting for within-subject correlations between two eyes of the sample patients^29^. Chi-square tests were used to assess the statistical significance of association between two categorical variables. T-tests were used to assess the statistical significance of subject-level variables. Sensitivity analysis was conducted with the same statistical procedures described above for the small Kappa angle < 0.3 mm and large Kappa angle $\ge$ 0.3 mm. To account for the multiple comparisons issue, p values were adjusted by Benjamini–Hochberg procedure^30^. All analyses were conducted using R^31^. A *p* value < 0.05 is considered statistically significant.

## Results

### Sample characteristics in small and large-Kappa groups

Table 1 shows the characteristics of 169 eyes grouped by small (< 0.2 mm, 81 eyes) and large Kappa angle ($\ge$ 0.2 mm, 88 eyes). Males were slightly predominant in both groups, accounting for 60.5% in the small Kappa (S-Kappa) group and 59.1% in the large Kappa (L-Kappa) group. For myopia levels, the S-Kappa group consisted of 22.2% with low myopia, 49.4% with moderate myopia, and 28.4% with high myopia. Comparatively, the L-Kappa group had 12.5%, 55.7%, and 31.8% respectively, with no significant statistical differences between the groups (*p* = 0.246). Post-operative UDVA, measured as logMAR, showed minimal differences between the groups (*p* = 0.222). Pre-operative mean spherical equivalent (MSE) was −4.7 (± 1.96) for the S-Kappa group and −4.93 (± 1.71) for the L-Kappa group, showing no significant difference (*p* = 0.433). The post-operative MSE was similar between the groups, being 0.23 (± 0.6) for the S-Kappa and 0.2 (± 0.48) for the L-Kappa, with a non-significant difference (*p* = 0.774).

**Table 1:** Characteristics of the study sample according to the Kappa angle (n = 169).

Variable	S-Kappa (n = 81)	L-Kappa (n = 88)	*p*
Gender			.977
Female	32 (39.5)	36 (40.9)	
Male	49 (60.5)	52 (59.1)	
Myopia level			.246
Low myopia	18 (22.2)	11 (12.5)	
Moderate myopia	40 (49.4)	49 (55.7)	
High myopia	23 (28.4)	28 (31.8)	
Age	22.3 (4.5)	24.9 (5.8)	.001**
logMAR	−0.04 (0.04)	−0.05 (0.06)	.222
Pre-operative Kappa	0.1 (0.05)	0.33 (0.07)	< .001***
Pre-operative MSE	−4.7 (1.96)	−4.93 (1.71)	.433
Post-operative MSE	0.23 (0.6)	0.2 (0.48)	.774

Continuous variables were expressed as mean (standard deviation). Categorical variables (Gender and Myopia Level) were expressed as frequencies (percentages).

S-Kappa, small Kappa group; L-Kappa, large Kappa group; MSE, mean spherical error; logMAR, logarithm of the minimum angle of resolution. *P*, *p* value from Chi-squared tests.

*for *p* value < .05, ** for *p* value < .01.

### Analysis of post-operative metrics between S-Kapp and L-Kappa angle groups across entire myopia spectrum

To compare post-operative metrics while accounting for within-subject correlations, we employed linear mixed models for all eyes including the entire spectrum of myopia. These models enabled us to determine the adjusted means, mean differences, and corresponding confidence intervals and p-values for each metric, as presented in Table 2. There was no significant difference in UDVA (logMAR adjusted mean difference: −0.01; 95% CI [−0.03, 0.01]) and postoperative MSE (adjusted mean difference: 0.03; 95% CI [−0.17, 0.23]). For a pupil size of 3 mm, there were statistically significant adjusted mean differences between the small and large Kappa groups in several parameters. Specifically, the Strehl ratio showed a difference of -0.06 (95% CI [−0.11, −0.02], *p* = 0.022), total HOA of 0.15 (95% CI [0.05, 0.25], *p* = 0.022), Horizontal Coma of 0.04 (95% CI [0.01, 0.06], *p* = 0.042), and Vertical Coma of 0.03 (95% CI [0.01, 0.05], *p* = 0.022). Despite their statistical significance, these differences might not translate into clinical relevance. For a 6 mm pupil size, no significant difference was observed in the Strehl ratio, total HOA, and horizontal coma, except for vertical coma, which showed a difference of −0.08 (95% CI [−0.14, −0.02], *p* = 0.041). Spherical Aberrations showed no significant difference between the two Kappa groups for both 3 mm and 6 mm pupil sizes.

**Table 2:** Comparison of adjusted means of post-operative metrics, accompanied by 95% confidence intervals and mean differences, between low and high kappa angle groups for all eyes.

Variable	S-Kappa (n = 81)	L-Kappa (n = 88)	Difference	*p*
logMAR	−0.04 [−0.05, −0.02]	−0.05 [−0.06, −0.04]	−0.01 [−0.03, 0.01]	.257
Postoperative MSE	0.21 [0.06, 0.36]	0.24 [0.09, 0.39]	0.03 [−0.17, 0.23]	.757
Strehl ratio (3 mm)	0.39 [0.36, 0.42]	0.32 [0.29, 0.35]	−0.06 [−0.11, −0.02]	.022*
Strehl ratio (6 mm)	0.17 [0.14, 0.19]	0.18 [0.15, 0.2]	0.01 [−0.03, 0.04]	.757
HOA (3 mm)	0.62 [0.54, 0.69]	0.77 [0.69, 0.84]	0.15 [0.05, 0.25]	.022*
HOA (6 mm)	0.77 [0.69, 0.84]	0.85 [0.78, 0.92]	0.08 [−0.01, 0.18]	.180
Horizontal Coma (3 mm)	0.06 [0.04, 0.08]	0.09 [0.07, 0.12]	0.04 [0.01, 0.06]	.042*
Horizontal Coma (6 mm)	0.17 [0.14, 0.2]	0.16 [0.13, 0.19]	−0.01 [−0.05, 0.03]	.757
Vertical Coma (3 mm)	0.06 [0.05, 0.08]	0.1 [0.08, 0.11]	0.03 [0.01, 0.05]	.022*
Vertical Coma (6 mm)	0.28 [0.23, 0.32]	0.2 [0.15, 0.24]	−0.08 [−0.14, −0.02]	.041*
Spherical aberrations (3 mm)	0.05 [0.03, 0.08]	0.09 [0.06, 0.12]	0.04 [0, 0.07]	.084
Spherical aberrations (6 mm)	0.27 [0.24, 0.31]	0.24 [0.21, 0.28]	−0.03 [−0.07, 0.01]	.257

The reported means and the mean differences were computed from linear mixed models that adjusted for age, gender, and pre-operative MSE as covariates.

S-Kappa, small Kappa group; L-Kappa, large Kappa group; MSE, mean spherical error; logMAR, logarithm of the minimum angle of resolution. HOA, Higher-Order Aberrations; *P*, *p* value from linear mixed models.

*for *p* value < .05, ** for *p* value < .01.

### Stratified analysis of post-operative outcomes in each myopia level

A stratified analysis using a linear mixed model was conducted to investigate the postoperative outcomes between small and large Kappa angle groups across three predefined levels of myopia: low, moderate, and high. Within the low and moderate myopia categories, no significant differences in post-operative metrics were identified between the two Kappa angle groups, as presented in Tables 3 and 4. In contrast, for the high myopia group (see Table 5), several significant mean differences were found. The mean difference was −0.04 (95% CI = [−0.08, −0.01], *p* = 0.037) for UDVA, 0.07 (95% CI [0.02, 0.12], *p* = 0.030) for horizontal coma at a 3 mm pupil size, and −0.21 (95% CI [−0.31, −0.11], *p* = 0.001) for vertical coma at a 6 mm pupil size. Additionally, spherical aberrations also exhibited statistically significant mean differences at both 3 mm (0.07, 95% CI = [0.01, 0.12], *p* = 0.037) and 6 mm pupil sizes (−0.09, 95% CI = [−0.16, −0.02], *p* = 0.037). While these differences are statistically significant, their clinical impact might be negligible.

**Table 3:** Comparison of adjusted post-operative metrics, accompanied by 95% confidence intervals and mean differences, between low and high Kappa angle groups for low myopia eyes.

Variable	S-Kappa (n = 18)	L-Kappa (n = 11)	Difference	*p*
logMAR	−0.06 [−0.1, −0.02]	−0.11 [−0.15, −0.06]	−0.04 [−0.08, 0]	.274
Postoperative MSE	0.02 [−0.36, 0.39]	0.15 [−0.23, 0.52]	0.13 [−0.27, 0.53]	.970
Strehl ratio (3 mm)	0.41 [0.31, 0.51]	0.41 [0.31, 0.51]	0 [−0.1, 0.1]	.970
Strehl ratio (6 mm)	0.19 [0.11, 0.27]	0.17 [0.09, 0.25]	−0.02 [−0.1, 0.06]	.970
HOA (3 mm)	0.53 [0.29, 0.76]	0.55 [0.3, 0.8]	0.02 [−0.22, 0.27]	.970
HOA (6 mm)	0.61 [0.41, 0.82]	0.62 [0.41, 0.83]	0.01 [−0.21, 0.22]	.970
Horizontal Coma (3 mm)	0.1 [0.04, 0.16]	0.11 [0.04, 0.17]	0.01 [−0.06, 0.07]	.970
Horizontal Coma (6 mm)	0.18 [0.09, 0.27]	0.26 [0.17, 0.35]	0.09 [0, 0.18]	.274
Vertical Coma (3 mm)	0.06 [0.01, 0.11]	0.11 [0.05, 0.16]	0.04 [−0.01, 0.1]	.367
Vertical Coma (6 mm)	0.2 [0.08, 0.32]	0.2 [0.08, 0.33]	0.01 [−0.12, 0.13]	.970
Spherical Aberrations (3 mm)	0.03 [−0.03, 0.09]	0.05 [−0.01, 0.11]	0.02 [−0.05, 0.09]	.970
Spherical Aberrations (6 mm)	0.23 [0.15, 0.31]	0.24 [0.16, 0.32]	0.02 [−0.07, 0.1]	.970

The reported means and the mean differences were computed from linear mixed models that adjusted for age, gender, and pre-operative MSE as covariates.

S-Kappa, small Kappa group; L-Kappa, large Kappa group; MSE, mean spherical error; logMAR, logarithm of the minimum angle of resolution. HOA, Higher-Order Aberrations; *P*, *p* value from linear mixed models.

*for *p* value < .05, ** for *p* value < .01.

**Table 4:** Comparison of adjusted post-operative metrics, accompanied by 95% confidence intervals and mean differences, between low and high kappa angle groups for moderate myopia eyes.

Variable	Low-Kappa (n = 40)	High-Kappa (n = 49)	Difference	*p*
logMAR	−0.05 [−0.07, −0.03]	−0.04 [−0.06, −0.02]	0.01 [−0.02, 0.03]	.744
Postoperative MSE	0.28 [0.08, 0.48]	0.32 [0.14, 0.5]	0.04 [−0.21, 0.3]	.744
Strehl ratio (3 mm)	0.39 [0.34, 0.43]	0.32 [0.28, 0.36]	−0.07 [−0.13, −0.01]	.097
Strehl ratio (6 mm)	0.16 [0.12, 0.19]	0.17 [0.13, 0.2]	0.01 [−0.04, 0.05]	.744
HOA (3 mm)	0.63 [0.52, 0.73]	0.81 [0.71, 0.9]	0.18 [0.04, 0.32]	.097
HOA (6 mm)	0.76 [0.66, 0.86]	0.88 [0.79, 0.97]	0.12 [−0.01, 0.26]	.174
Horizontal Coma (3 mm)	0.05 [0.03, 0.08]	0.08 [0.06, 0.11]	0.03 [−0.01, 0.07]	.288
Horizontal Coma (6 mm)	0.19 [0.15, 0.24]	0.13 [0.1, 0.17]	−0.06 [−0.11, −0.01]	.097
Vertical Coma (3 mm)	0.07 [0.04, 0.09]	0.1 [0.08, 0.12]	0.04 [0, 0.07]	.097
Vertical Coma (6 mm)	0.25 [0.19, 0.31]	0.2 [0.15, 0.26]	−0.04 [−0.12, 0.03]	.506
Spherical Aberrations (3 mm)	0.06 [0.03, 0.1]	0.08 [0.05, 0.11]	0.02 [−0.02, 0.06]	.592
Spherical Aberrations (6 mm)	0.25 [0.2, 0.29]	0.23 [0.19, 0.27]	−0.02 [−0.07, 0.04]	.744

The reported means and the mean differences were computed from linear mixed models that adjusted for age, gender, and pre-operative MSE as covariates.

S-Kappa, small Kappa group; L-Kappa, large Kappa group; MSE, mean spherical error; logMAR, logarithm of the minimum angle of resolution. HOA, Higher-Order Aberrations; *P*, *p* value from linear mixed models.

*for *p* value < .05, ** for *p* value < .01.

**Table 5:** Comparison of adjusted post-operative metrics, accompanied by 95% confidence intervals and mean differences, between low and high kappa angle groups for high myopia eyes.

Variable	Low-Kappa (n = 23)	High-Kappa (n = 28)	Difference	*p*
logMAR	0 [−0.04, 0.03]	−0.04 [−0.07, −0.01]	−0.04 [−0.08, −0.01]	.037*
Postoperative MSE	0.23 [−0.08, 0.55]	0.15 [−0.11, 0.41]	−0.08 [−0.41, 0.24]	.665
Strehl ratio (3 mm)	0.37 [0.3, 0.45]	0.29 [0.23, 0.36]	−0.08 [−0.16, 0]	.086
Strehl ratio (6 mm)	0.17 [0.11, 0.23]	0.2 [0.14, 0.25]	0.03 [−0.04, 0.09]	.499
HOA (3 mm)	0.66 [0.47, 0.85]	0.79 [0.61, 0.96]	0.13 [−0.06, 0.31]	.317
HOA (6 mm)	0.88 [0.72, 1.05]	0.9 [0.76, 1.05]	0.02 [−0.15, 0.19]	.809
Horizontal Coma (3 mm)	0.04 [−0.01, 0.09]	0.11 [0.07, 0.15]	0.07 [0.02, 0.12]	.030*
Horizontal Coma (6 mm)	0.13 [0.06, 0.2]	0.17 [0.11, 0.23]	0.04 [−0.03, 0.11]	.383
Vertical Coma (3 mm)	0.06 [0.01, 0.1]	0.08 [0.04, 0.12]	0.02 [−0.02, 0.06]	.439
Vertical Coma (6 mm)	0.38 [0.28, 0.48]	0.17 [0.09, 0.26]	−0.21 [−0.31, −0.11]	.001**
Spherical Aberrations (3 mm)	0.06 [0, 0.11]	0.12 [0.08, 0.17]	0.07 [0.01, 0.12]	.037*
Spherical Aberrations (6 mm)	0.35 [0.28, 0.41]	0.26 [0.2, 0.31]	−0.09 [−0.16, −0.02]	.037*

The reported means and the mean difference were computed from a linear mixed model that adjusted for age, gender, and pre-operative MSE as covariates.

S-Kappa, small Kappa group; L-Kappa, large Kappa group; MSE, mean spherical error; logMAR, logarithm of the minimum angle of resolution. HOA, Higher-Order Aberrations; P, p value from linear mixed models.

*for *p* value < .05, ** for *p* value < .01.

### Sensitivity analysis

To assess the potential impact of varying Kappa angle thresholds on our results, we conducted a sensitivity analysis using a threshold of 0.3 mm to define S-Kappa and L-Kappa groups. Upon this examination, no statistically significant differences were noted in post-operative wave aberrations, UDVA, or MSE between the S-Kappa and L-Kappa groups (Supplemental Tables S1–S5).

## Discussion

In this study, we examined the mean differences in postoperative UDVA, measured in logMAR, MSE, SR, total HOAs, MSE HC, VC, and SA among eyes with small and large Kappa angles. To diminish the influence of the kappa angle on eccentric ablation, we systematically incorporated a Kappa angle adjustment procedure on all eyes before suction initiation, as outlined by Shao et al.^14^. This repositioning of the treatment center towards the visual axis facilitated a decrease in induced coma magnitude.

The significance of the Kappa angle in refractive surgeries has been a focal point of extensive research^15,17,32^. Within our cohort of eyes with mild to moderate myopia, we found no significant difference between groups with small and large Kappa angles. However, for the high myopia subgroup, a statistically significant difference was observed between the small and large Kappa angle groups in UDVA, HC, VC, and SA. Notably, postoperative logMAR, VC (6 mm), and SA (6 mm) for the L-Kappa group were significantly lower than those in the S-Kappa group, indicating superior outcomes. However, the HC (3 mm) and SA (3 mm) of the L-Kappa group exceeded those of the S-Kappa group. Despite statistical significance, these differences lacked clinical relevance. In an analysis involving all eyes, SR, total HOA, HC, and VC were significantly different between the S-Kappa and L-Kappa groups. However, despite a larger sample size potentially offering a more robust model, the variations between the S-Kappa and L-Kappa angle groups remained clinically insignificant.

Xie et al., 2023, reported no statistically significant difference in wavefront aberrations between S-Kappa (< 0.3 mm) and L-Kappa (≥ 0.3 mm) groups from a cohort of 12 patients^33^. The VC, for instance, was -0.03 and -0.026 for the S-Kappa and L-Kappa groups, respectively. While a small sample size could potentially limit the power to detect minor effect sizes, the use of arithmetic means over root square means could counteract both positive and negative wavefront aberration values, leading to underestimation of the wavefront aberrations. Although our results demonstrated a statistically significant difference between S-kappa and L-kappa groups using a 0.2 mm threshold, no substantial difference was found when using a 0.3 mm kappa angle threshold. In a separate study, Shao et al. compared postoperative ocular aberrations in eyes with large Kappa angle, with or without intraoperative Kappa angle adjustment^14^. They found a significant correlation in the non-adjusted group, but not in the adjusted group, suggesting the importance of adjusting the Kappa angle during SMILE. Our sensitivity analysis results align with this report by Shao et al., showing no significant difference between the large and small kappa groups when both receive kappa adjustment. Zhang et al. (2023) observed that despite a significant increase in optical aberrations post-SMILE (for instance, a 0.25 increase in total HOAs), over 96% of patients reported equal or superior visual quality compared to preoperative assessments^34^. Therefore, the observed differences in optical aberrations between groups in our study are not clinically significant.

This study also adds to the body of literature by stratifying results based on the degree of myopia. Several studies have investigated the outcomes of SMILE or LASIK surgeries across different degrees of myopia^32,35–37^. Our findings align with theirs, demonstrating the efficacy of SMILE across various myopia levels. However, these studies have not addressed whether large Kappa angles affect SMILE outcomes. Our results indicate that a large Kappa angle may not significantly impact post-operative optical aberrations when intraoperative Kappa angle adjustments are utilized. Interestingly, we observed differences in post-operative metrics based on the kappa angle for patients with high myopia. This suggests that the kappa angle could play a more substantial role in patients with high myopia, underlining the need for careful pre-operative consideration and planning for these patients.

Our study has several limitations. First, its retrospective design might be prone to selection bias. Secondly, although the Kappa angle was determined using a reputable and broadly endorsed methodology, the possibility of measurement inaccuracies can never be entirely ruled out. The sample of patients was also obtained from a single center, potentially constraining the wider applicability of our findings. Therefore, further investigations are recommended to explore the variations in clinical outcomes and optical aberrations between postoperative patients with small and large Kappa groups.

Our study is distinguished using linear mixed models (LMM) in our analysis of multilevel data. This approach, superior to conventional methods like t-tests or analysis of variance (ANOVA), offers several benefits. These include the ability to account for within-subject correlations often neglected in traditional analyses, the flexibility to adjust for both fixed and random effects, and providing more accurate parameter estimates^38^. To the best of our knowledge, this study is the first to employ the LMM approach in evaluating postoperative optical aberrations between different Kappa angle groups.

Using LMM offers several benefits. First, they are capable of accounting for within-subject correlations, which is often overlooked in traditional methods. This capability recognizes the potential influence of individual-specific characteristics and ensures that the statistical inference is more precise. Secondly, linear mixed models allow for the inclusion of covariates, which provides a more comprehensive understanding of the influence of potential confounding variables^39^. This aspect further enhances the reliability and validity of the results, making linear mixed models a superior choice for data analysis in our study.

## Conclusions

In conclusion, this study evaluated the differences in optical aberrations between S-Kappa and L-Kappa groups. It found no statistically significant difference when using a Kappa angle threshold of 0.3 mm. When a threshold of 0.2 mm was applied, though statistical significance was observed in the high myopia group, the differences in optical aberrations and visual acuity were not clinically significant. Therefore, the study suggests that with intraoperative Kappa angle adjustment, optical aberrations and acuity may not be significantly affected in patients with large Kappa angles.

### Supplementary Information

Supplementary Information 1.

Supplementary Information 2.

## Data Availability

The datasets used and/or analyzed during the current study are available from the corresponding author on reasonable request.
