# Supplementary material for: Comparison of visual outcomes and optical aberrations after SMILE with intraoperative Kappa angle adjustments between small and large Kappa angles
Source: Sci Rep. 2024 Jun 24;14:14551. doi: 10.1038/s41598-024-65366-w (PMC11196622; doi:10.1038/s41598-024-65366-w)
Supplement: Supplementary file 1 — Supplementary Information 1. [file 41598_2024_65366_MOESM1_ESM.docx]

Supplemental table 1. Characteristics of the study sample according to the Kappa angle (n = 169).

| Variable | S-Kappa (n=106) | L-Kappa (n=63) | *p* |
| --- | --- | --- | --- |
| Gender |  |  | .961 |
| Female | 42 (39.6) | 26 (41.3) |  |
| Male | 64 (60.4) | 37 (58.7) |  |
| Myopia level |  |  | .123 |
| Low myopia | 23 (21.7) | 6 (9.5) |  |
| Moderate myopia | 52 (49.1) | 37 (58.7) |  |
| High myopia | 31 (29.2) | 20 (31.7) |  |
| Age | 22.9 (4.9) | 24.8 (6) | .024* |
| logMAR | -0.04 (0.05) | -0.05 (0.05) | .300 |
| Pre-operative Kappa | 0.13 (0.08) | 0.36 (0.05) | < .001*** |
| Pre-operative MSE | -4.73 (1.95) | -4.98 (1.63) | .394 |
| Post-operative MSE | 0.24 (0.57) | 0.18 (0.49) | .534 |

Note: Continuous variables were expressed as mean (standard deviation). Categorical variables (Gender and Myopia Level) were expressed as frequencies (percentages).

S-Kappa, small Kappa group; L-Kappa, large Kappa group; MSE, mean spherical error; logMAR, logarithm of the minimum angle of resolution. P, p value from Chi-squared tests.

* for p value less than .05, ** for p value less than .01.

Supplemental table 2. Adjusted means of post-operative metrics, accompanied by 95% confidence intervals and mean differences, between low and high kappa angle groups for all eyes.

| Variable | S-Kappa (n=81) | L-Kappa (n=88) | Difference | *p* |
| --- | --- | --- | --- | --- |
| logMAR | -0.04 [-0.05 , -0.03] | -0.05 [-0.06 , -0.03] | -0.01 [-0.02, 0.01] | .758 |
| Postoperative MSE | 0.24 [0.12 , 0.36] | 0.21 [0.07 , 0.35] | -0.03 [-0.17, 0.11] | .758 |
| Strehl ratio (3mm) | 0.36 [0.33 , 0.39] | 0.35 [0.31 , 0.38] | -0.02 [-0.06, 0.02] | .758 |
| Strehl ratio (6mm) | 0.17 [0.15 , 0.19] | 0.18 [0.15 , 0.2] | 0.01 [-0.02, 0.04] | .758 |
| HOA (3mm) | 0.67 [0.61 , 0.74] | 0.73 [0.64 , 0.81] | 0.06 [-0.04, 0.16] | .635 |
| HOA (6mm) | 0.8 [0.74 , 0.86] | 0.83 [0.75 , 0.9] | 0.03 [-0.05, 0.11] | .758 |
| Horizontal Coma (3mm) | 0.07 [0.05 , 0.09] | 0.09 [0.07 , 0.11] | 0.02 [0, 0.05] | .251 |
| Horizontal Coma (6mm) | 0.17 [0.14 , 0.19] | 0.17 [0.14 , 0.2] | 0 [-0.03, 0.04] | .820 |
| Vertical Coma (3mm) | 0.07 [0.06 , 0.09] | 0.09 [0.08 , 0.11] | 0.02 [0, 0.05] | .251 |
| Vertical Coma (6mm) | 0.26 [0.22 , 0.29] | 0.2 [0.15 , 0.24] | -0.06 [-0.1, -0.01] | .251 |
| Spherical Aberrations (3mm) | 0.07 [0.05 , 0.09] | 0.08 [0.05 , 0.1] | 0 [-0.02, 0.03] | .758 |
| Spherical Aberrations (6mm) | 0.26 [0.24 , 0.29] | 0.24 [0.21 , 0.28] | -0.02 [-0.05, 0.01] | .561 |

Note: The reported means and the mean difference were computed from a linear mixed model that adjusted for age, gender, and pre-operative MSE as covariates.

S-Kappa, small Kappa group; L-Kappa, large Kappa group; MSE, mean spherical error; logMAR, logarithm of the minimum angle of resolution. HOA, Higher-Order Aberrations; P, p value from linear mixed models.

* for p value less than .05, ** for p value less than .01.

Supplemental table 3. Adjusted means post-operative metrics, accompanied by 95% confidence intervals and mean differences, between low and high kappa angle groups for low myopia eyes.

| Variable | S-Kappa (n=18) | L-Kappa (n=11) | Difference | *p* |
| --- | --- | --- | --- | --- |
| logMAR | -0.08 [-0.12 , -0.04] | -0.09 [-0.13 , -0.04] | -0.01 [-0.05, 0.04] | .948 |
| Postoperative MSE | 0.05 [-0.29 , 0.39] | 0.14 [-0.26 , 0.54] | 0.09 [-0.29, 0.46] | .948 |
| Strehl ratio (3mm) | 0.42 [0.32 , 0.51] | 0.44 [0.32 , 0.56] | 0.02 [-0.09, 0.13] | .948 |
| Strehl ratio (6mm) | 0.19 [0.12 , 0.26] | 0.16 [0.07 , 0.25] | -0.03 [-0.12, 0.06] | .948 |
| HOA (3mm) | 0.51 [0.28 , 0.74] | 0.5 [0.21 , 0.79] | -0.01 [-0.29, 0.27] | .955 |
| HOA (6mm) | 0.62 [0.43 , 0.81] | 0.57 [0.35 , 0.8] | -0.05 [-0.26, 0.17] | .948 |
| Horizontal Coma (3mm) | 0.09 [0.04 , 0.15] | 0.11 [0.04 , 0.18] | 0.01 [-0.05, 0.08] | .948 |
| Horizontal Coma (6mm) | 0.18 [0.1 , 0.26] | 0.3 [0.2 , 0.4] | 0.12 [0.03, 0.22] | .181 |
| Vertical Coma (3mm) | 0.08 [0.03 , 0.13] | 0.08 [0.01 , 0.15] | 0 [-0.06, 0.07] | .955 |
| Vertical Coma (6mm) | 0.22 [0.11 , 0.34] | 0.2 [0.06 , 0.34] | -0.02 [-0.15, 0.11] | .948 |
| Spherical Aberrations (3mm) | 0.04 [-0.02 , 0.1] | 0.03 [-0.04 , 0.09] | -0.01 [-0.07, 0.05] | .948 |
| Spherical Aberrations (6mm) | 0.26 [0.18 , 0.33] | 0.22 [0.13 , 0.3] | -0.04 [-0.12, 0.04] | .948 |

Note: The reported means and the mean difference were computed from a linear mixed model that adjusted for age, gender, and pre-operative MSE as covariates.

S-Kappa, small Kappa group; L-Kappa, large Kappa group; MSE, mean spherical error; logMAR, logarithm of the minimum angle of resolution. HOA, Higher-Order Aberrations; P, p value from linear mixed models.

* for p value less than .05, ** for p value less than .01.

Supplemental table 4. Adjusted means post-operative metrics, accompanied by 95% confidence intervals and mean differences, between low and high kappa angle groups for moderate myopia eyes.

| Variable | S-Kappa (n=40) | L-Kappa (n=49) | Difference | *p* |
| --- | --- | --- | --- | --- |
| logMAR | -0.04 [-0.06 , -0.02] | -0.04 [-0.06 , -0.02] | 0 [-0.03, 0.02] | .829 |
| Postoperative MSE | 0.27 [0.11 , 0.43] | 0.32 [0.14 , 0.49] | 0.05 [-0.14, 0.23] | .801 |
| Strehl ratio (3mm) | 0.37 [0.33 , 0.41] | 0.33 [0.29 , 0.38] | -0.04 [-0.09, 0.02] | .437 |
| Strehl ratio (6mm) | 0.15 [0.12 , 0.18] | 0.17 [0.14 , 0.2] | 0.02 [-0.03, 0.06] | .801 |
| HOA (3mm) | 0.68 [0.58 , 0.77] | 0.78 [0.67 , 0.89] | 0.1 [-0.03, 0.23] | .437 |
| HOA (6mm) | 0.78 [0.7 , 0.86] | 0.87 [0.78 , 0.96] | 0.09 [-0.02, 0.19] | .414 |
| Horizontal Coma (3mm) | 0.07 [0.04 , 0.09] | 0.08 [0.05 , 0.1] | 0.01 [-0.02, 0.04] | .801 |
| Horizontal Coma (6mm) | 0.18 [0.15 , 0.21] | 0.14 [0.1 , 0.18] | -0.04 [-0.09, 0] | .414 |
| Vertical Coma (3mm) | 0.07 [0.05 , 0.09] | 0.1 [0.08 , 0.13] | 0.03 [0, 0.06] | .414 |
| Vertical Coma (6mm) | 0.25 [0.19 , 0.3] | 0.2 [0.15 , 0.26] | -0.04 [-0.11, 0.02] | .437 |
| Spherical Aberrations (3mm) | 0.06 [0.04 , 0.09] | 0.07 [0.05 , 0.1] | 0.01 [-0.02, 0.04] | .801 |
| Spherical Aberrations (6mm) | 0.24 [0.2 , 0.27] | 0.24 [0.2 , 0.27] | 0 [-0.04, 0.03] | .829 |

Note: The reported means and the mean difference were computed from a linear mixed model that adjusted for age, gender, and pre-operative MSE as covariates.

S-Kappa, small Kappa group; L-Kappa, large Kappa group; MSE, mean spherical error; logMAR, logarithm of the minimum angle of resolution. HOA, Higher-Order Aberrations; P, p value from linear mixed models.

* for p value less than .05, ** for p value less than .01.

Supplemental table 5. Adjusted means post-operative metrics, accompanied by 95% confidence intervals and mean differences, between low and high kappa angle groups for high myopia eyes.

| Variable | S-Kappa (n=23) | L-Kappa (n=28) | Difference | *p* |
| --- | --- | --- | --- | --- |
| logMAR | -0.02 [-0.05 , 0.01] | -0.04 [-0.07 , -0.01] | -0.02 [-0.05, 0.01] | .326 |
| Postoperative MSE | 0.28 [0.02 , 0.54] | 0.08 [-0.17 , 0.34] | -0.2 [-0.42, 0.03] | .326 |
| Strehl ratio (3mm) | 0.31 [0.24 , 0.39] | 0.34 [0.26 , 0.41] | 0.02 [-0.05, 0.09] | .661 |
| Strehl ratio (6mm) | 0.18 [0.12 , 0.23] | 0.19 [0.14 , 0.25] | 0.02 [-0.04, 0.07] | .661 |
| HOA (3mm) | 0.77 [0.59 , 0.95] | 0.71 [0.53 , 0.9] | -0.06 [-0.23, 0.11] | .661 |
| HOA (6mm) | 0.93 [0.79 , 1.08] | 0.86 [0.71 , 1] | -0.08 [-0.21, 0.05] | .409 |
| Horizontal Coma (3mm) | 0.06 [0.01 , 0.1] | 0.11 [0.07 , 0.16] | 0.06 [0.01, 0.1] | .121 |
| Horizontal Coma (6mm) | 0.14 [0.08 , 0.2] | 0.18 [0.12 , 0.25] | 0.05 [-0.01, 0.1] | .326 |
| Vertical Coma (3mm) | 0.06 [0.02 , 0.1] | 0.07 [0.03 , 0.12] | 0.01 [-0.03, 0.05] | .730 |
| Vertical Coma (6mm) | 0.29 [0.2 , 0.38] | 0.2 [0.11 , 0.29] | -0.09 [-0.17, -0.01] | .190 |
| Spherical Aberrations (3mm) | 0.1 [0.05 , 0.14] | 0.1 [0.06 , 0.14] | 0 [-0.04, 0.04] | .953 |
| Spherical Aberrations (6mm) | 0.31 [0.25 , 0.36] | 0.27 [0.21 , 0.32] | -0.04 [-0.08, 0.01] | .326 |

Note: The reported means and the mean difference were computed from a linear mixed model that adjusted for age, gender, and pre-operative MSE as covariates.

S-Kappa, small Kappa group; L-Kappa, large Kappa group; MSE, mean spherical error; logMAR, logarithm of the minimum angle of resolution. HOA, Higher-Order Aberrations; P, p value from linear mixed models.

* for p value less than .05, ** for p value less than .01.
